# Expression of Selected Connexin and Aquaporin Genes and Real-Time Proliferation of Porcine Endometrial Luminal Epithelial Cells in Primary Culture Model

**DOI:** 10.1155/2020/7120375

**Published:** 2020-02-03

**Authors:** Katarzyna Wojtanowicz-Markiewicz, Magdalena Kulus, Sandra Knap, Ievgenia Kocherova, Maurycy Jankowski, Katarzyna Stefańska, Michal Jeseta, Hanna Piotrowska-Kempisty, Dorota Bukowska, Maciej Zabel, Paul Mozdziak, Michał Nowicki, Bartosz Kempisty, Paweł Antosik

**Affiliations:** ^1^Veterinary Centre, Nicolaus Copernicus University in Torun, Torun, Poland; ^2^Department of Histology and Embryology, Poznan University of Medical Sciences, Poznan, Poland; ^3^Department of Anatomy, Poznan University of Medical Sciences, Poznan, Poland; ^4^Department of Obstetrics and Gynaecology, University Hospital and Masaryk University, Brno, Czech Republic; ^5^Department of Toxicology, Poznan University of Medical Sciences, Poznan, Poland; ^6^Division of Histology and Embryology, Department of Human Morphology and Embryology, Wroclaw Medical University, Wroclaw, Poland; ^7^Division of Anatomy and Histology, University of Zielona Góra, Zielona Góra, Poland; ^8^Physiology Graduate Program, North Carolina State University, Raleigh, North Carolina, USA

## Abstract

Luminal epithelial cells are the first embryonic-maternal contact site undergoing very specific changes associated with reproductive processes. Cells prepare for embryo development by increasing their volume, with the help of aquaporins that provide a transcellular path of rapid water movement during the secretion and absorption of fluids, as well as connexins enabling the flow of inorganic ions and small molecules. In this work, we have examined how AQPs and Cx's behave in luminal epithelium primary cell culture. Cells obtained from porcine specimen during slaughter were primarily in vitro cultured for 7 days. Their proliferation patterns were then analyzed using RTCA, with the expression of genes of interest evaluated with the use of immunofluorescence and RT-qPCR. The results of these changes of gene of interest expression were analyzed on each of the seven days of the porcine luminal primary cell culture. Our study showed that the significant changes were noted in the case of Cx43, whose level of protein expression and distribution increases after 120 hours of culture, when the cells enter the lag phase, and maintains an upward trend until the end of the culture. We noted an increase in AQP4, AQP7, AQP8, and AQP11 levels throughout the entire culture period, while the largest differences in expression were found in AQP3, AQP4, and AQP10. The obtained results could become a point of reference for further in vivo and clinical research. Experiments conducted with these proteins showed that they influence the endometrial fluid content during the oestrous cycle and participate in the process of angiogenesis, which intensifies during endometrial development.

## 1. Introduction

Continuing the cycle of research on gene expression in the porcine luminal epithelium cells in this work, we have tested the expression of genes responsible for the distribution of proteins involved in cellular communication. These involved gap junction channel connexins: Cx36 (GJD2-gap junction protein delta 2, Cx36 protein), Cx37 (GJA4-gap junction protein alpha 4, Cx37 protein), Cx40 (GJD4-gap junction protein delta 4, Cx40 protein), and Cx43 (GJA1-gap junction protein alpha 1, Cx43 protein), as well as simple water channels in the form of aquaporins: AQP2, AQP3, AQP4, AQP5, AQP6, AQP7, AQP8, AQP 9, AQP 10, and AQP11.

Connexins (GJC) are a large family of proteins that form gap junction; a single connexin forms hexagonal structures called connexons (nexus type connections). Connexons of two adjacent cells form a gap junction connection [1, 2]. The construction of the gap junctions varied, depending on the structure of individual connexons. These can be made of one type of proteins and combine with the other connexons also built from the same type of proteins creating a homomeric-homotypic connection. We also distinguish the homomeric-heterotypic connection in which different proteins build connexons on both of the cells participating in gap junction. In turn, the heterometric-homotypic connection is characterized by a combination of connexon with a uniform protein structure and a connexon of diverse protein structure. There is also a combination with the biggest protein structure diversity, in which both cones are made of various proteins called heteromeric heterotypic connections. The protein structure of individual connexons is responsible for the biophysical properties of gap junction connections [1, 3].

Gap junctions allow for extremely fast transfer of electrical signal between cells, as well as a slower flow of ions, small molecules, and secondary relays [1, 2]. In 1990 studies conducted on the human uterine epithelium, changes in gap junction configuration were observed during the menstrual cycle. The size and distribution of gap junctions in human endometrial epithelial cells varied depending on the phases of the menstrual cycle. It results with synchronization of proliferation and differentiation of the endometrial epithelium regulated by the by gap junction mediated cellular adherence. In the early phase of the proliferation, gap junctions were few and of small size, in contrast to the early secretory phase in which the connections were larger and more frequent [4].

Abnormal expression and localization of gap junction proteins in these connections may cause problems during embryo implantation and reproductive abnormalities. For example, conditional deletion of several different transcription factors alters the receptivity of the uterus by interfering with the expression of the claudin-1 tight junction protein. In addition, the reduced function of connexin 43 through dominant loss of function or administration of pharmacological inhibitor interferes with early implantation events, in particular lyophilization and early placental angiogenesis, with severe consequences for fetal health [5, 6].

Aquaporins (AQPs) are a large family of water channel proteins located on membranes of various cell types, where they contribute to high water permeability and provide a transcellular path of rapid movement during the secretion and absorption of fluid. The connections are formed by two highly conserved regions in the amino acid sequence, called NPA boxes (or motifs) with three amino acid residues (asparagine-proline-alanine, NPA) and several surrounding amino acids. The NPA boxes have been called the “signature” sequence of aquaporins [7, 8].

Aquaporins are integral transmembrane channel proteins. They are primarily responsible for water transport, regulating the normal state of cellular homeostasis. For this protein family, we can distinguish three subtypes. The first type, constituting AQP0, AQP1, AQP2, AQP4, AQP5, AQP6, and AQP8, consists of classical aquaporins and selective channels permeable to water but not to small organic and inorganic molecules. The second type allows for the transport of glycerine and small molecules dissolved in water and contains, e.g., AQP3, AQP7, AQP9, and AQP10 [9]. Such a structure allows for selectivity of the substance transport, thus protecting the cells against hydronium ion penetration. The third type, superaquaporins, having poorly conserved asparagine-proline-alanine (NPA) boxes, consists of AQP11 and AQP12 [7, 10, 11].

During implantation, an increased number of aquaporin channels (AQP2) were noted in the uterine epithelium. It has been proved that the synergistic effect of aquaporins influence changes in endometrial fluid content during the oestrous cycle. Experiments conducted with these proteins also showed their participation in the process of angiogenesis, which intensify during the phase of the endometrial development and embryo implantation [11, 12]. Recent studies in humans with AQP gene mutations as well as those using transgenic AQP knockout mice indicate that AQPs play an important role in the physiology and pathophysiology. To date, at least 12 different proteins from the AQP family have been located in various mammalian tissues [10, 11].

In this work, we focused on 10 proteins from the AQP fluid transporter family and the 4 best-known connexins so far found in the endometrium. The main aim of the manuscript was to investigate their expression during in vitro culture and relate the results to the data obtained during real-time proliferation assay. The obtained results should provide information about the formation of the protein connections of interest, serving to improve knowledge about the in vitro processes occurring in the endometrial epithelial cells. Additionally, the data obtained might serve as a basic reference for further in vivo and clinical studies.

## 2. Materials and Methods

### 2.1. Animals

In this study, crossbred gilts (*n* = 45) at the age of about nine months and which displayed two regular oestrous cycles were collected from a commercial herd. After slaughter, their uteri were then transported to the laboratory within 30 min at 38°C. The samples were combined to obtain the necessary number of living cells to perform the test.

### 2.2. Endometrial Cell (EC) Selection and Culture

After transport, the uterine horns were washed twice with PBS (137 mM NaCl, 27 mM KCl, 10 mM Na_2_HPO_4_, 2 mM KH_2_PO_4_ (pH 7.4)), with small pieces immediately being cut out (25–35 mg) from the middle uterine horn. Surface uterine epithelial cells were removed using sterile surgical blades. The cell suspension obtained from this digestion was filtered through a mesh to remove nondissociated fragments of tissue and incubated with 0.05% collagenase I (Sigma-Aldrich, Madison, USA) for 25 min at 38°C in a shaking water bath. Isolated cells were washed three times by centrifugation (10 min at 200 g) with Dulbecco's modified Eagle's medium (DMEM; Sigma-Aldrich, Madison, USA) supplemented with gentamicin (20 *μ*g/mL) and 0.1% BSA. The resulting luminal epithelial cells were filtered and washed three times with supplemented DMEM by centrifugation (10 min at 200 g). The final pellet of luminal epithelial cells was resuspended in DMEM supplemented with 10% fetal calf serum (FCS; Sigma-Aldrich, Madison, USA) and 10,000 U mL^−1^ penicillin G, 10 mg mL^−1^ streptomycin, and 25 *μ*g mL^−1^ amphotericin B (antibiotic antimycotic solution; Sigma-Aldrich, Madison, USA). The viability of the cells was 90 to 95%, as judged by trypan blue staining (Sigma-Aldrich, Madison, USA). The cells were cultured at 38°C in a humidified atmosphere of 5% CO_2_. The culture medium was changed every 3 days [13, 14].

### 2.3. In Vitro Endometrial Cell Culture Using a Real-Time Cell Analyzer (RTCA)

The recovered endometrial cells were transferred into an E-Plate 16 in a real-time cell analyzer (RTCA, Roche-Applied Science, GmbH, Penzberg, Germany), which consisted of an RTCA analyzer, an RTCA SP station, and RTCA software. For each of the 16 wells was plated 50,000-cell collective sample. The cells were then cultured in 200 *μ*l of standard porcine culture medium that consisted of Dulbecco's modified Eagle's medium (DMEM, Sigma-Aldrich, USA) and supplements listed above. The cells were cultured for 0–168 h at 38.5° C under 5% CO_2_ in air. The cultivation medium was replaced every three days. After each period of cultivation, the cells were treated with trypsin (0.25% trypsin in a balanced salt solution, Sigma-Aldrich, St. Louis, MO, USA), with the collected pool of proliferating cells used for RT-qPCR analysis. After each culture period, the cell index (CI) was used to evaluate the relative and quantitative changes in electrical cell impedance. The cell status was determined using RTCA software [15, 16].

### 2.4. Confocal Microscopy Analysis of Connexin Expression and Distribution in Porcine Luminal Epithelial Cells

The luminal epithelial cells were transferred into an X-well Lumox container (94.6150.401, 4-well Lumox Detachable, Sarstedt AG & Co. KG). The cells were then cultured in 1 ml of standard porcine culture medium that consisted of Dulbecco's modified Eagle's medium (DMEM, Sigma-Aldrich, USA) and supplements mentioned above. The cells were cultured for 0–168 h at 38.5°C under 5% CO_2_. The cultivation medium was replaced every three days. The cells were fixed using acetone-methanol (1 : 1) for 10 min at −20°C and washed three times in PBS/PVP (0.2%). To block nonspecific binding, samples were incubated in 3% BSA in PBS with 0.1% Tween 20 for 30 min at RT. The luminal epithelial cells were incubated for 1 hour at room temperature (RT) with mouse monoclonal anti-Cx36/GJD2 antibody (sc-398063), goat polyclonal anti-Cx37/GJA4 antibody (sc-27711), rabbit polyclonal anti-Cx43/GJA1 antibody (sc-271837; Santa Cruz Biotechnology, Santa Cruz, CA, USA), and rabbit polyclonal anti-Cx40/GJD4 antibody (ab38580; Abcam, Cambridge Biomedical Campus, Cambridge, UK), all diluted 1 : 500 in PBS/1.5% BSA/0.1% Tween 20. After several washes with PBS/0.1% Tween 20, samples treated with primary antibodies mentioned above were incubated for 1 hour at RT with fluorescein isothiocyanate- (FITC-) conjugated goat anti-rabbit IgG Ab, diluted 1 : 500 in PBS/0.1% Tween 20. Following washing in PBS/0.1% Tween 20, the luminal epithelial cells were stained with 0.1 *μ*g/ml 4,6-diamino-2-phenylindole (DAPI; Santa Cruz Biotechnology, Santa Cruz, CA, USA) in mineral oil, mounted on glass slides in an antifade solution drop, and examined under an LSN 510 confocal system in a Fluoview 10i microscope (Olympus). FITC was excited at 488 nm with an argon laser, with emissions being imaged through a 505–530 nm filter. All confocal microscopic images were analyzed using Imaris 7.2 software (BitPlane, Zurich, Switzerland) [13, 14]. In order to validate the specificity of antibodies for all samples, we used control experiment where only secondary antibody was used, and to analyze the changes in the expression of proteins, we used 10 images for each sample.

### 2.5. RNA Extraction from Porcine Luminal Epithelial Cells

Total RNA was extracted from samples using TRI Reagent (Sigma, St. Louis, MO, USA) and RNeasy MinElute clean-up Kit (Qiagen, Hilden, Germany). The amount of total mRNA was determined from the optical density at 260 nm, and the RNA purity was estimated using the 260/280 nm absorption ratio (higher than 1.8) (Nano Drop spectrophotometer, Thermo Scientific, ALAB, Poland). The RNA integrity and quality were checked on a Bioanalyzer 2100 (Agilent Technologies, Inc., Santa Clara, CA, USA). The resulting RNA integrity numbers (RINs) were between 8.5 and 10 with an average of 9.2 (Agilent Technologies, Inc., Santa Clara, CA, USA). The RNA in each sample was diluted to a concentration of 100 ng/*μ*l with an OD260/OD280 ratio of 1.8/2.0. From each RNA sample, 500 ng of RNA was taken for the RT-qPCR study [8].

### 2.6. Real-Time Quantitative Polymerase Chain Reaction (RT-qPCR) Analysis

Total RNA was isolated from luminal epithelial cells each of the seven days of culture. The RNA samples were resuspended in 20 *μ*l of RNase-free water and stored in liquid nitrogen. RNA samples were treated with DNase I and reverse-transcribed (RT) into cDNA. RT-qPCR was conducted in a Light Cycler real-time PCR detection system (Roche Diagnostics GmbH, Mannheim, Germany) using SYBR® Green I as a detection dye, with target cDNA quantified using the relative quantification method. The relative abundance of AQP2, AQP3, AQP4, AQP5, AQP6, AQP7, AQP8, AQ9, AQP10, and AQP11 and Cx36, Cx37, Cx40, and Cx43 transcripts in each sample was standardized to the internal standards. For amplification, 2 *μ*l of cDNA solution was added to 18 *μ*l of QuantiTect® SYBR® Green PCR (Master Mix Qiagen GmbH, Hilden, Germany) and primers (Table 1). One RNA sample of each preparation was processed without the RT-reaction to provide a negative control for subsequent PCR, where each sample processes in two technical repeats [17–19].

### 2.7. Statistical Analysis

One-way ANOVA followed by the Tukey post hoc test was used to compare the real-time (RTCA) quantification results. The experiments were carried out in at least four replicates. The differences were considered to be significant at ^*∗*^*P* < 0.05, ^*∗∗*^*P* < 0.01, and ^*∗∗∗*^*P* < 0.001. The statistical calculations were applied to compare the results in each investigated group to the highest normalized proliferation index at each time point. GraphPad Prism version 4.0 software (GraphPad Software, San Diego, CA) was used for all statistical calculations.

## 3. Results

**Figure 1:**
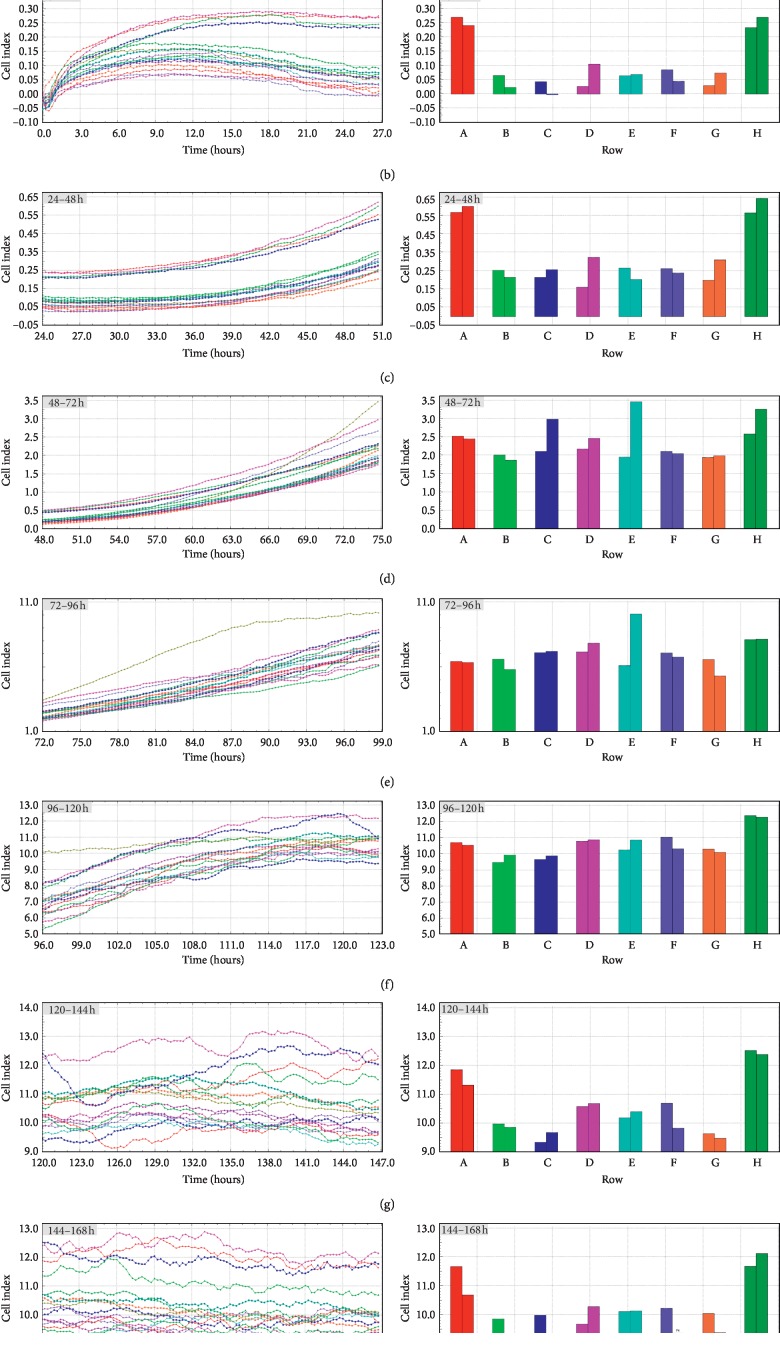
Cell proliferation index (CI) of porcine luminal epithelial cells cultivated for 168 h. The experiment consisted of eight replicates involving the cultivation of the same population of collected cells. The cell proliferation index (CI) was assessed for real-time in vitro cultivation for the time periods 0–24 h (a), 24–48 h (b), 48–72 h (c), 72–96 h (d), 96–120 h (e), 120–144 h (f), and 144–168 h (g). CI is an unitless parameter calculated based on impedance of electron flow caused by adherent cells; CI = (impedance at time point *n* − impedance in the absence of cell)/nominal impedance value. The differences were considered to be significant at the level of ^*∗*^*P* < 0.05, ^*∗∗*^*P* < 0.01, and ^*∗∗∗*^*P* < 0.001.

**Figure 2:**
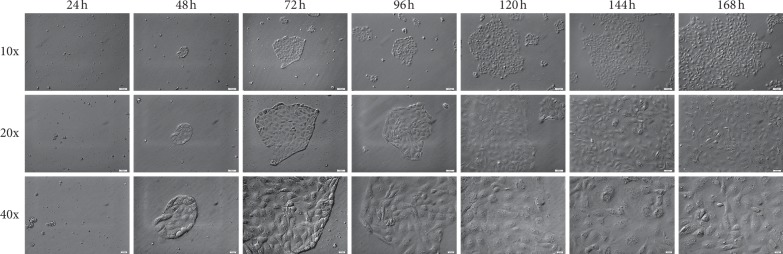
Representative picture of changes in cell morphology during short-term (168 h) primary cell culture. The cells isolated from porcine luminal epithelium cell were cultured for 168 h. Each 24 h cells growth and correct morphology changes were documented by Nomarski system with 10x, 20x, and 40x objective lenses (total magnifications: 6.3x, 12.6x, and 25.2x, resp.; M total = M objective × M video camera  adapter).

RTCA analysis showed a specific pattern of cell proliferation based on cell index analysis. The cell index was almost unchanged for the first 51 hours. Later, an increase was observed. Around hour 120, the cell index settled at a similar constant level. The total increase in cell index varied between the cell samples. However, every population's index increased. The results of specific time periods were presented in Figure 1. In turn, Figure 2, the pictures show that the correct morphological development of luminal epithelium cells, thanks to which we can exclude errors resulting from poorly developing cell culture cells analyzed during RTCA, were subjected to immunofluorescent staining, using specific antibodies for Cx36, Cx37, Cx40, and Cx43.

**Figure 3:**
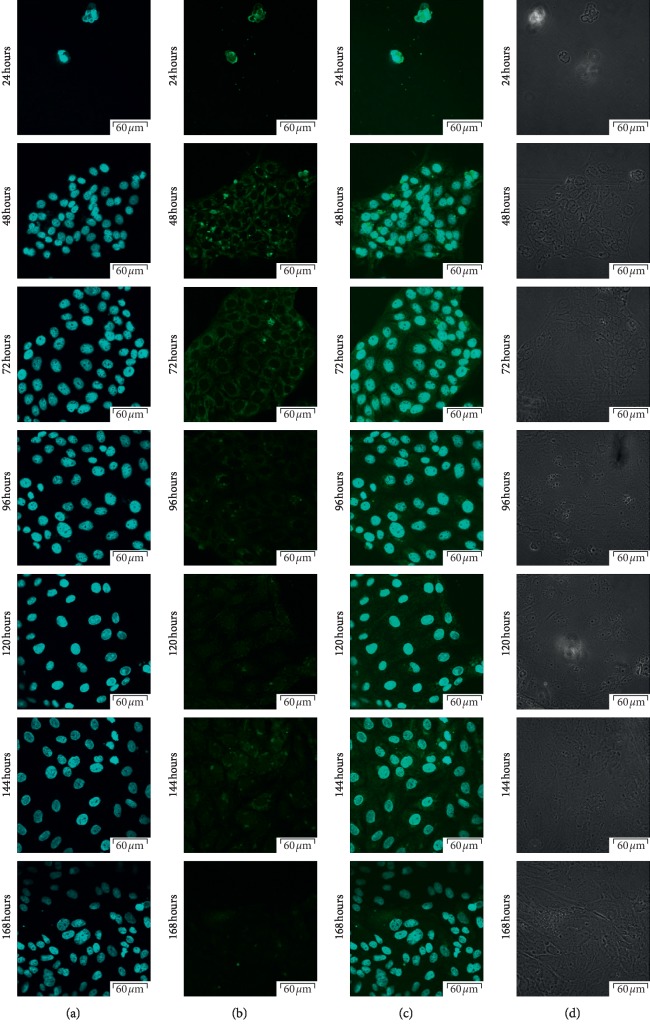
Photos of immunofluorescence test results, with the primary antibody being designed to detect Cx36 (GJD2-gap junction protein delta 2). (a) Nuclear staining with DAPI; (b) results of Cx36 detection using FITC; (c) positive control using DAPI, presented together with Cx36 detection using FITC; (d) phase contrast.

**Figure 4:**
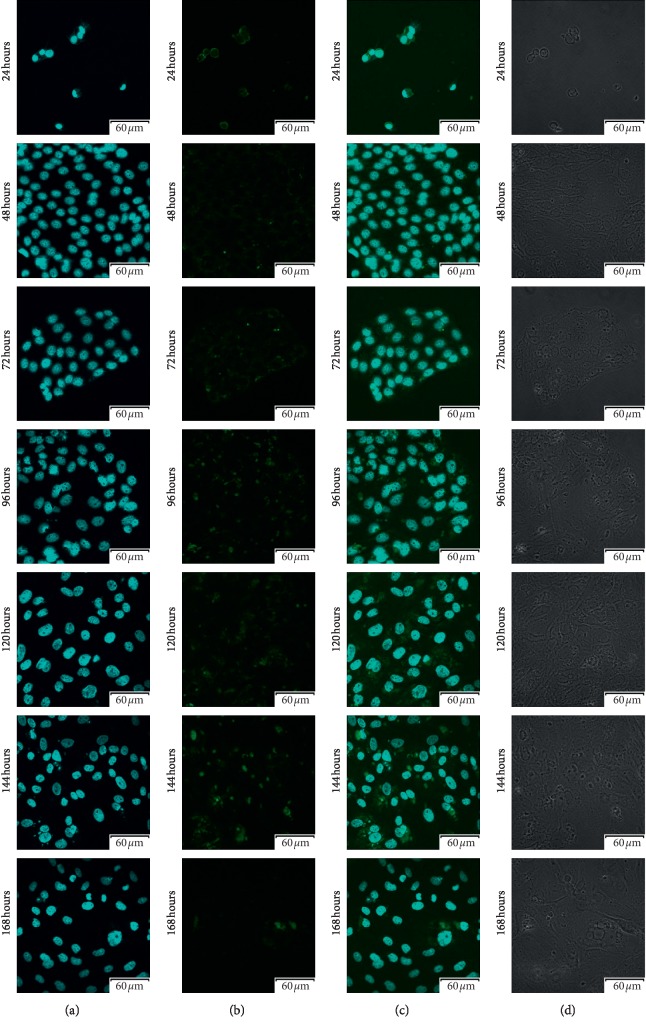
Photos of immunofluorescence test results, with the primary antibody being designed to detect Cx37 (GJA4-gap junction protein alpha 4). (a) Nuclear staining with DAPI; (b) results of Cx37 detection using FITC; (c) positive control using DAPI, presented together with Cx37 detection using FITC; (d) phase contrast.

**Figure 5:**
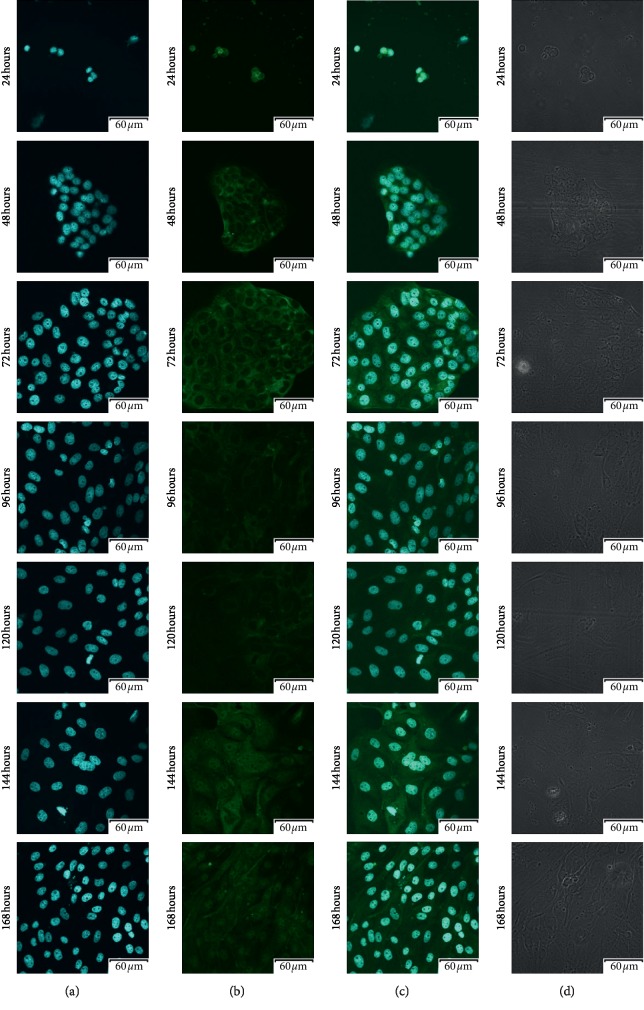
Photos of immunofluorescence test results, with the primary antibody being designed to detect Cx40 (GJD4-gap junction protein delta 4). (a) Nuclear staining with DAPI; (b) results of Cx40 detection using FITC; (c) positive control using DAPI, presented together with Cx40 detection using FITC; (d) phase contrast.

**Figure 6:**
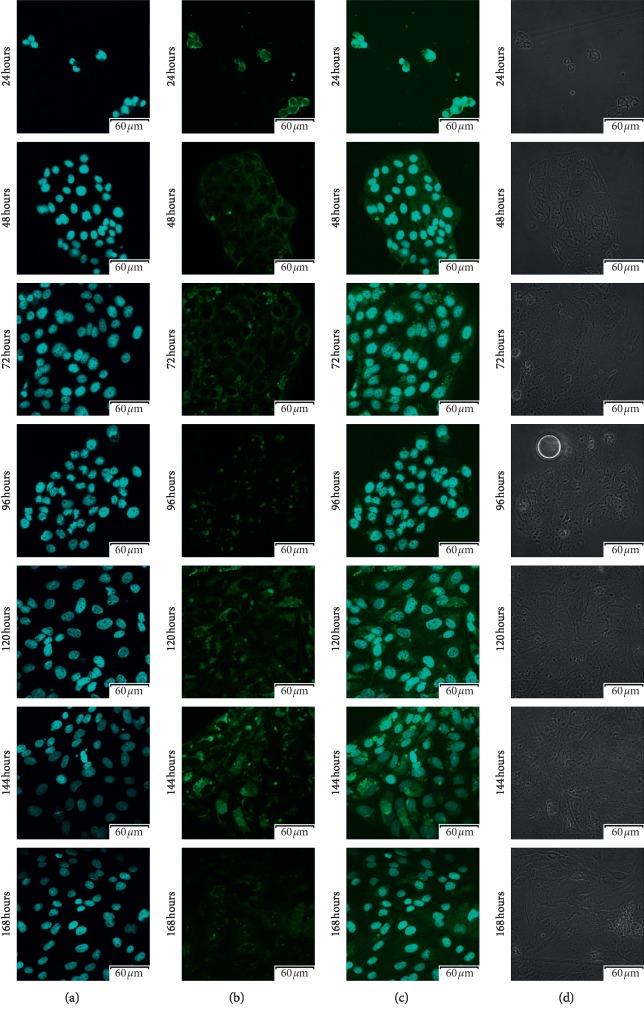
Photos of immunofluorescence test results, with the primary antibody being designed to detect Cx43 (GJA1-gap junction protein alpha 1). (a) Nuclear staining with DAPI; (b) results of Cx43 detection using FITC; (c) positive control using DAPI, presented together with Cx43 detection using FITC; (d) phase contrast.

Using confocal microscopic observation, we analyzed the expression and cellular distribution in each 7-day cell culture. The results of that analysis were presented in Figures 3–6, respectively. As can be seen, intense intracellular and extranuclear luminosity can be observed in all of the tests and time periods apart from the 24th hour of culture. While in that case, the fluorescence is not observable easily, it is present, as confirmed by the fluorescence graph.

**Figure 7:**
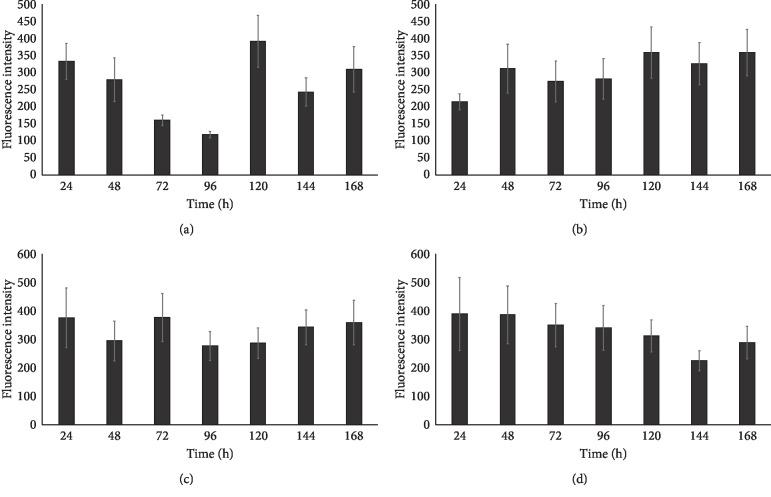
Region of interest (ROI) fluorescence intensity measurement results, presented as a bar graph for different stages of primary culture. (a) Cx36. (b) Cx37. (c) Cx40. (d) Cx43.

**Figure 8:**
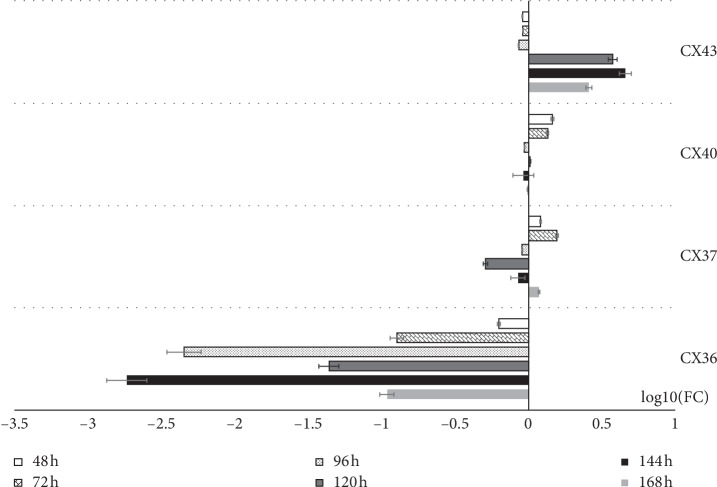
The results of RT-qPCR (real-time-quantitative polymerase chain reaction) validation of analyzed genes, presented in the form of bar graph. All the fold changes were described in relation to the transcript levels at 24 h of primary culture. LogFC was used to present the data in order to improve the clarity and comparability of upregulation and downregulation results. Connexins 36 (GJA1-gap junction protein alpha), 37 (GJA4-gap junction protein alpha 4), 40 (GJA1-gap junction protein alpha), and 43 (GJA1-gap junction protein alpha 1).

**Figure 9:**
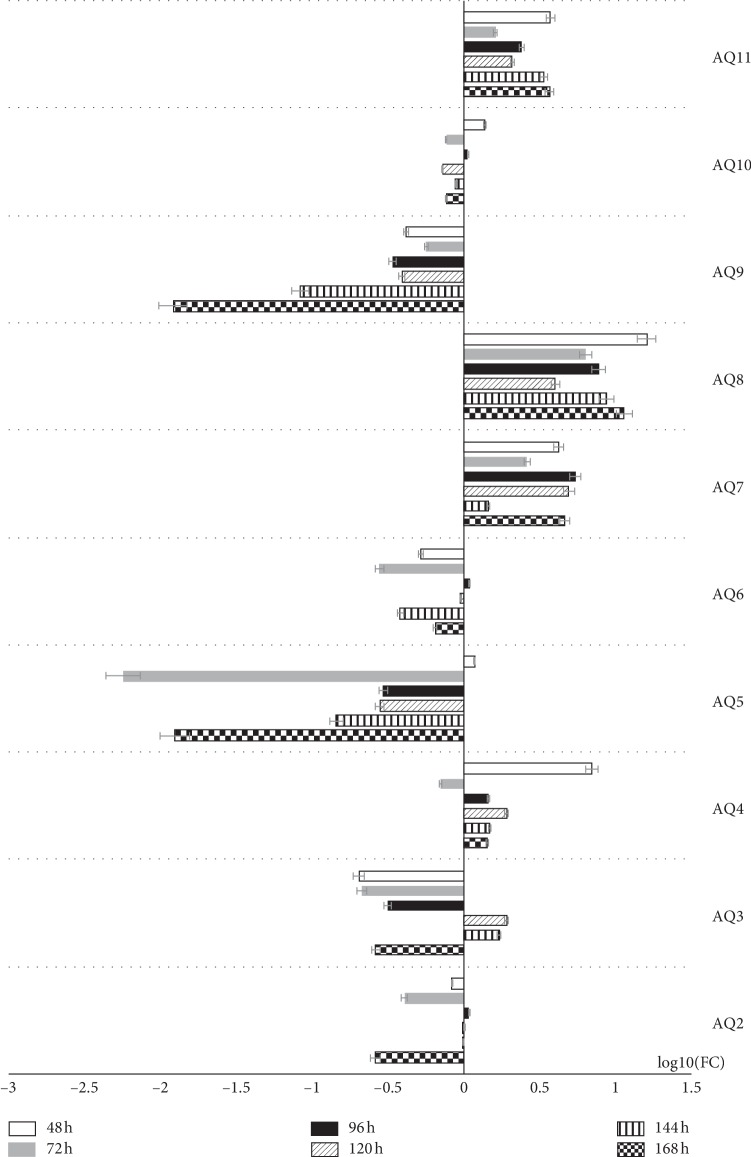
The results of RT-qPCR (real-time-quantitative polymerase chain reaction) validation of analyzed genes, presented in the form of bar graph. All the fold changes were described in relation to the transcript levels at 24 h of primary culture. LogFC was used to present the data in order to improve the clarity and comparability of upregulation and downregulation results. Aquaporin: AQP2, AQP3, AQP4, AQP5, AQP6, AQP7, AQP8, AQP9, AQP10, and AQP11.

These results confirm the presence of all of the analyzed cellular antigens throughout the culture period. As seen on the graphs (Figure 7), most of the markers present distinct patterns of fluorescence intensity. The immunofluorescence of Cx36 is low until hour 96, with a later increase peaking at hour 120. At hour 144, we recorded significant decrease in the intensity of expression of this protein, followed by another increase at 168 hours that lasted until the end of observation period. Throughout the observation period, Cx37 immunofluorescence continuously increased, and only at 48 and 120 hours, there were slight variations in the fluorescence intensity. The expression was similarly distributed on Cx40 protein, the lowest fluorescence intensity was recorded at the 48 and 96 hours of the study, and the highest at 24, 72, and 168 hours, and we observed that the immunofluorescent at the 120 hours was increased. Throughout the observation period, Cx43 immunofluorescence decreased until 144 hours. Then, at 168 hours, an increase in fluorescence intensity was noted. The highest intensity of fluorescence was recorded in the first hours of the study, with peak at 24 hours. Finally, the levels of the studied markers were analyzed using the RT-qPCR method. The results were presented in a form of a bar graph (Figures 8 and 9).

As can be seen, the highest level of the expression of the Cx36 gene was recorded at 24 hours of culture, and Cx37's expression peaked at 72 hours, with the expression of Cx40 reaching the highest levels at hours 96 and 144 and the expression of Cx43 reaching the highest levels at 144 hours of culture. Cx37, Cx40, and Cx43 transcript levels were increased relative to 24 h of culture throughout the study period. The levels of the Cx37 transcript were elevated at 48, 72, and 168 hours of culture, with the same pattern being observed for Cx40 at 48 and 72 hours. In addition, the Cx43 transcript level remains higher than the initial point of culture, with a notable increase at hour 120, when the cells enter the lag phase, maintaining a strong upward trend until the end of the culture. The results were presented in a form of a bar graph (Figure 8).

In the results of the RT-qPCR method, we observe a similar trend in AQP2 and AQP6 expressions. The highest level of expression is observed at 96 hours, with the level of expression of these genes being lower than the initial culture point throughout the other culture periods. As can be seen, AQP7, AQP8, and AQP11 maintained an elevated level of expression throughout the culture. In each of these three cases, the level of expression at 72 hours decreases slightly, followed by a subsequent increase. However, in the case of AQP8 and AQP11, the highest level of expression is recorded at 48 hours of observation. In turn, the transcript levels of the AQP5 and AQP10 genes are lowered during almost the entire culture period with only the highest level of expression at 48h of culture exceeding the initial transcript level. As can be seen in the case of AQP9, the highest transcript level is reached at 24 h of culture and remains lower than the initial value throughout the rest of the culture period. In turn, the highest level of AQP4 expression was recorded at the 48 hour of culture. Then, the transcript levels drop significantly (below the 24 h level). In the next hours of culture, the levels of expression increase, again exceeding those observed at the start of the culture, staying at similar levels until the end of culture period. As can be seen, AQP3 reaches the highest level of gene expression at hours 120 and 144. Only during this period, the transcript level was higher than that observed during the initial culture hours. The results were presented in a form of a bar graph (Figure 9).

## 4. Discussion

Our previous results indicated the expression and possible function of Cx's in pig GCs during short-term primary culture [6]. In this study, porcine endometrial epithelium cells were used as a research model, in which the expression pattern of Cx's (Cx36, Cx37, Cx40, and Cx43) was evaluated during short-term real-time in vitro culture (IVC). The ultrastructure of human endometrial epithelial cells shows marked structural changes during the menstrual cycle. A role in this process is attributed to gap junctions, the diversified area of the plasma membrane of neighbouring cells allowing the substance to diffuse from the cell into the cell through the low resistance pathway. In the epithelium, cyclic AMP and other small molecules can pass through gap junction junctions and play an important role in coordinating the reactions of epithelial cell groups to hormonal and nervous stimuli [6, 20].

Research on selectivity and permeability of ions of various sizes, charges, and chemical compositions, conducted using fluorescent labels, revealed a wide range of pore properties. Molecules other than ions are large enough to be excluded from passage, and homotypic combinations of Cx43, Cx40, Cx37, and Cx45 junctions show similar selectivity to monovalent cations (e.g., K^+^ and Na^+^). However, selectivity of charge varies from low anionic preference (Cx32) to high cationic selectivity (Cx40 and Cx43). Channel behaviour is usually complex, involving the main fully open state as well as multiple intermediate states of varying selectivity. The uniform conductivity of the gap junction channels depends on the connexin isotypes. The Cx36 channels exhibit a unit conductivity of 14 pS, while the Cx37 channels have a very high electrical conductivity of 300 pS [6, 21]. The role of oestrogen and progesterone in the expression of Cx in various types of ovarian and uterine tissues has recently been studied in several mammalian species. However, the expression profile of Cx in endometrial epithelium cells is unknown [22].

The role of Cx's in the regulation of oocyte growth and development as well as differentiation of cumulus cells through GJC-mediated induction is widely recognized. It is also known that this process is regulated by factors, e.g., hormones secreted in a paracrine manner. However, mechanisms regulating GJC activity and/or communication pathways between luminal epithelium cells still need to be explored [23, 24].

Studies conducted on adult ovariectomized rats injected with 20 *μ*g of 17*β*-estradiol (E2) or sesame oil 48 hours before sacrifice and further injected with 1.5 mg of progesterone or sesame oil 24 hours before killing, using Northern blot as a detection method, revealed that oestrogen significantly increases connexin-36 mRNA expression in SCN (suprachiasmatic nucleus), with progesterone inhibiting that process. However, the connexin-36 mRNA level was not altered by oestrogen or progesterone alone. These results suggest that the combination of the connexin-36 junctions in SCN is specifically regulated by the steroid hormones of female rats [25, 26]. In our studies, we tested the mRNA expression of selected connexins, such as Cx36, Cx37, Cx40, and Cx43, with RT-qPCR; the distribution of Cx36, Cx37, Cx40, and Cx43 proteins in porcine luminal epithelial cells during short-term culture (168 h); and real-time in vitro cell proliferation. We observed increased expression of Cx37 and Cx40 mRNA in the first two periods (24, 48 h, *P* < 0.001), as well as that of Cx43 in the last three periods (120, 144, and 168 h, *P* < 0.001) of luminal epithelial cell culture, compared to all other analyzed IVC periods (24, 48, 72, 96, 120, 144, and 168 h, *P* < 0.001). In addition, the expression of Cx36 and Cx37 proteins was higher before the culture (0 h), compared to 168h of IVC. This may indicate a gradual reduction/“leaching” of hormones from the cells to which they were exposed in the body. Similarly, the highest increase of the PI (proliferation index) value was seen between 56 and 96 hours of IVC. Considering both results, we suggest that the reduced expression of Cx and/or breakdown of GJC during IVC can be significantly associated with cell differentiation and apoptosis in vitro [6, 25]. Our research was based on a monoculture model of luminal epithelial cells originating from a primary 2D culture, which did not include enrichment of the culture medium with ingredients that could stimulate the growth and development of these cells. Thanks to that, we obtained an image of the possible gap junction connections created by these cells in vitro. Our research identified the expression of AQP2–11 by the means of RT-qPCR. However, the level of gene expression in relation to the first day of cell culture, which we adopted as the starting level, was shaped in different ways [27, 28].

The level of AQP4, AQP7, AQP8, and AQP11 transcripts throughout the cell culture period was maintained above the starting level. As reported by Richard et al., AQP4 are located only in the luminal epithelium on days 1 and 4 of pregnancy, with less presence on day 4. There were no specific AQP4 signals in the glandular epithelium nor near the implanting blastocyst. Distributed signals were recorded in the uterine stroma on day 5, but AQP4 probes showed a similar pattern, suggesting nonspecific hybridization [12, 28].

Limited AQP8 mRNA expression was reported in embryonic endoderm and mesometrial decidualizing stroma in the site of future placenta development. AQP8 was also expressed in retreating glands of the uterus during the exfoliation phase. Low levels of AQP9 accumulation were localized in the mesometrial decidua at this time but were not present in the developing embryo. Studies showing different locations in implantable blastocysts and placenta development sites suggest that AQP8 and AQP9 have distinct functions in cellular turgidity or fluid/soluble molecule transport during embryonic-placental development [27, 29].

The expression of the AQP5 mRNA was restricted to the glandular epithelium on days 1, 4, 4.5, and 5 of pregnancy. Compared to the modest expression on D1 an intensive accumulation of AQP5 signals in glands was observed on day 5. Longitudinal sections that included the implantation and interimplantation regions (data not shown) revealed the same pattern of AQP5 expression in the glandular epithelium along the length of the uterus. It demonstrated that the AQP5 transcripts were not limited to the site of implantation. In previous studies, we suggested the possibility of luminal cells transdifferentiation into cells of mesenchymal characteristics, which could explain the presence of AQP5 on the first day of culture [30, 31].

We know that the expression of AQP2 in endometrial luminal epithelial cells is dependent on the menstrual cycle and endometrial glands, suggesting that E2 can regulate the expression of AQP2 in endometrial cells. In addition, our group showed that estrogen/estrogen receptor complex may bind to the AQP2 gene promoter region and promote AQP2 gene expression in endometrial cells. High endometrial expression of AQP2 during the proliferative and cell differentiation phases suggests that AQP2 may contribute to endometrial susceptibility for embryo implantation [12, 28, 32]. AQP3 was also detected in the uterine epithelial cells. The distribution of AQP2 and AQP3 suggests that both of them contribute to the movement of water in the lumen of the uterus [9]. In our research, we only observe proteins responsible for the transport in cells without additional hormonal stimulation. Hence, we can observe the potential of cells to express individual proteins [30, 33].

Overall, the aim of this study was to study the expression of aquaporins, as well as and the cellular and subcellular locations of connexins in porcine endometrial luminal epithelial cells. Considering the level of expression of all AQPs and connexins tested in the first 24 hours of culture, we assume that these cells exhibit both epithelial and stromal/glandular properties in order to maintain optimal conditions for the development of cellular monoculture. In this study, we examined the abundance of mRNA and proteins responsible for communication between apertures and the development of luminal epithelium cells. We have shown that specific levels of transcript and protein can be associated with the morphology of luminal epithelium cells. In addition, we demonstrated that the expression of the junctional genes has a specific location in the cytoplasm depending on the day of cell culture, which may affect the normal development of the endometrium, acquisition of cytoplasmic competence, and normal physiological functions of luminal epithelium. Therefore, the levels of fissure junction proteins in the luminal epithelium cells can be an important indicator of endometrium development.

## Figures and Tables

**Table 1:** Primer information and primer sequences used for the RT-qPCR analysis.

Gene	3′–5′	5′–3′
*AQP2*	ccccgccctctccattggtt	ctccagccccttcagcaccg
*AQP3*	cctggatcaagctgcccgtct	cgaagaagccattgaccatgtcc
*AQP4*	cagtatgaaccctgcccgat	atccgcctccatgtagctc
*AQP5*	gcaatctggccgtcaactcg	cggtgaagtagatccccacaagg
*AQP6*	ccggctgctccatgaaccc	cactttctccacctccgcagt
*AQP7*	cgtctactgccttttctatggtg	atgactacaaggatgccgat
*AQP8*	ccccattctccatcggctt	tctcagcttcaccgtccct
*AQP9*	tcacaggagaaaacgcaac	aaaacagcgaagactataatgag
*AQP10*	gacagcctccatctttgaccac	atcccaccatcagcaaccag
*AQP11*	ttcgcttgcaggaatccca	gccaaagctggattaaataccg
*Cx36*	agcagcactccactatgatcgg	tgttgcacacaaacatggtct
*Cx37*	gtgtgtccgcaccgccaccgatga	acaggaccgtcagccaga
*Cx40*	atcgctttcacctgcaagtcc	cgccatcctcagcagaaccat
*Cx43*	cagcacttttctttcattaggggc	ctaaggactccagtcacc
*ACTB*	cccttgccgctccgccttc	gcagcaatatcggtcatccat
*HPRT*	ccatcacatcgtagccctc	acttttatatcgcccgttgac

## Data Availability

The data used to support the findings of this study are available from the corresponding author upon request.
